# Biosecurity Practices in Portuguese Small Ruminant Farms: Current Status and Future Directions

**DOI:** 10.3390/vetsci12040334

**Published:** 2025-04-04

**Authors:** Maria Alavedra, Dina Moura, Beniamino Cenci-Goga, Sónia Saraiva, Filipe Silva, Isabel Pires, Cristina Saraiva, Ana Cláudia Coelho, Juan García-Díez

**Affiliations:** 1 Veterinary and Animal Research Centre (CECAV), University of Trás-os-Montes e Alto Douro, Quinta de Prados, 5000-801 Vila Real, Portugal; mariadebourbonalavedra@gmail.com (M.A.); soniasaraiva@utad.pt (S.S.); fsilva@utad.pt (F.S.); ipires@utad.pt (I.P.); crisarai@utad.pt (C.S.); acoelho@utad.pt (A.C.C.); 2 Department of Veterinary Sciences, School of Agricultural and Veterinary Sciences, Universidade de Trás-os-Montes e Alto Douro, Quinta de Prados, 5000-801 Vila Real, Portugal; 3 Divisão de Intervenção de Alimentação e Veterinária de Vila Real e Douro Sul, Direção de Serviços de Alimentação e Veterinária da Região Norte, Direção Geral de Alimentação e Veterinária, Lugar de Codessais, 5000-421 Vila Real, Portugal; dina.moura@dgav.pt; 4 Dipartimento di Medicina Veterinaria, Università degli Studi di Perugia, 06126 Perugia, Italy; beniamino.cencigoga@unipg.it; 5 Faculty of Veterinary Science, Department of Paraclinical Sciences, University of Pretoria, Onderstepoort 0110, South Africa; 6 Associate Laboratory for Animal and Veterinary Science (AL4AnimalS), 1300-477 Lisboa, Portugal

**Keywords:** small ruminants, sheep, goat, biosecurity, compliance, farm

## Abstract

Biosecurity is essential in livestock farming to prevent the spread of diseases, ensure animal welfare, and maintain farm sustainability. In Portugal, small ruminant farms are predominantly extensive and small-scale, and most of them are familiar, especially in the northern and inland regions. Thus, biosecurity implementation on these farms is low due to factors such as an aging livestock farmer population, poor training, limited veterinary support, and economic constraints. This study, the first to assess biosecurity on Portuguese small ruminant farms, evaluated compliance levels and the influence of sociodemographic factors. A cross-sectional study was conducted between July 2023 and April 2024, through structured interviews with 276 farmers. A 32-question checklist covering nine biosecurity categories was used to assess compliance. The results revealed poor implementation of key biosecurity measures, particularly cleaning and disinfection, quarantine protocols, and visitor control. Inadequate premises infrastructure, including the absence of quarantine areas, isolation facilities for sick animals, and farrowing rooms, further hampered disease prevention. Compliance was influenced by farmers’ age, education level, herd size, and production. Larger farms, particularly dairy farms, demonstrated better biosecurity practices, likely due to better management and infrastructure. This study highlights the challenges of implementing biosecurity measures on small-scale, extensive farms and argues that standardized plans are ineffective. Instead, region- and farm-specific strategies are needed, considering the socioeconomic realities of farmers. Improving farmers’ education and access to veterinary services is crucial. Furthermore, public policies should provide financial incentives and educational programs to improve biosecurity without compromising farm viability. Strengthening biosecurity on small ruminant farms is vital to protecting animal and public health and ensuring the long-term sustainability of rural communities in Portugal.

## Simple Summary

Biosecurity is crucial in livestock farming to prevent the spread of disease, ensure animal welfare, and maintain sustainability. In Portugal, small ruminant production is predominantly extensive, small-scale, and family-run. This study assessed biosecurity on 276 farms through a cross-sectional survey (July 2023 to April 2024), analyzing compliance with nine biosecurity categories. The results showed low implementation of key measures such as cleaning, disinfection, quarantine, and visitor control. Poor infrastructure further hampered disease prevention. Factors affecting compliance included farmers’ age, education, herd size, and production type, with dairy and larger farms performing best. This study highlights the need for training, veterinary support, and policies to improve biosecurity while maintaining farm viability and protecting public health in rural Portugal.

## 1. Introduction

Livestock production of small ruminants, such as sheep and goats, has a long-standing tradition in Portugal, primarily carried out on family farms with relatively small herds. In 2016, the average number of animals per farm was 49.5, reflecting the extensive nature of this agricultural activity. Small ruminant farming often complements other agricultural activities, providing economic stability and employment opportunities, particularly in rural areas [1,2,3]. According to the National Institute of Statistics of Portugal, the sheep population in 2019 was 2.2 million heads, distributed across approximately 43,000 farms, with the majority located in the Alentejo and Beira Interior regions. These regions account for about two-thirds of the national sheep population, with an average of 141.5 heads per farm, significantly higher than the national average of 51.1. This is due to the extensive areas of low agricultural use, which are ideal for free grazing systems [3]. In contrast, sheep production in northern Portugal is characterized by small-scale family farms with fewer animals, often relying on natural pastures and native breeds. Traditional practices remain prevalent in the north, partly due to the high average age of farmers.

Goat production in Portugal follows a similar pattern, with a population of around 372,000 heads distributed across 22,900 farms. The Alentejo region hosts 23% of the goat population, while the rest is evenly distributed across the North, Center, Ribatejo, and West regions. Goat herds are generally smaller than sheep herds, averaging 16.3 heads per farm [3]. Both sheep and goat farming play a crucial role in Portugal’s agricultural sector, particularly in less favored regions where other forms of agriculture are less economically viable [4,5]. These activities provide essential income and employment, supporting many rural households. Additionally, value-added products, such as PDO Portuguese cheeses like Queijo da Serra da Estrela, contribute to the sector’s profitability [6].

Despite their importance, sheep and goat production in Portugal face several challenges. The decreasing number of livestock, coupled with the high average age of farmers [3], poses a significant threat to the sector. Other contributing factors include limited farmer training, low technological adoption, increasing legal requirements, small herd sizes, and the persistence or reappearance of certain diseases. These issues collectively impact the economic performance of small ruminant farming [7].

One of the main challenges affecting profitability is herd health management. Veterinary support is often limited to official surveillance campaigns, such as those for brucellosis and blue tongue, with veterinarians typically contacted only when animals fall ill. Ensuring herd health is essential for profitability, making disease prevention measures critical [8]. The emergence of infectious diseases can severely jeopardize a farm’s economic viability, highlighting the importance of implementing biosecurity plans. Biosecurity can be defined as a set of management and physical measures designed to reduce the risk of introduction, establishment, and spread of animal diseases, infections, or infestations to, from (external biosecurity or bio-exclusion), and within (internal biosecurity or biocontainment) an animal population [9].

Developing a biosecurity plan requires the expertise of veterinarians experienced in pathogen control. Key aspects to consider include livestock movement, animal isolation (quarantine), and the sanitation of facilities. While biosecurity measures have been widely studied and implemented in intensive livestock systems, such as poultry and pig farming, their application in extensive ruminant production presents unique challenges. Extensive systems, common in Portugal, often involve grazing on communal lands, making it difficult to control environmental and management factors [10].

A biosecurity plan typically involves three phases: identifying potential hazards, assessing their impact, and evaluating the likelihood of introduction and spread. This approach is similar to the Hazard Analysis and Critical Control Points (HACCP) system used in the food industry. General biosecurity measures include facility and equipment management, animal movement control, health monitoring, feeding and watering practices, external vector control, visitor management, manure handling, and carcass disposal [11].

The implementation of biosecurity measures in ruminant farms has been the subject of several studies, both in bovine production [12,13,14] and in the production of small ruminants [14,15]. European legislation places the responsibility for animal health on livestock farmers, emphasizing the importance of biosecurity as a preventive tool. While the implementation of biosecurity plans may require upfront investments, the long-term benefits, such as reduced disease incidence [16,17], improved animal welfare, and compliance with regulations, can outweigh the costs [18]. Additionally, biosecurity measures protect public health [19] by controlling zoonotic diseases, safeguard the environment, and enhance market access.

Traditionally, biosecurity plans have been implemented in intensive livestock farms, such as poultry and pig farms, due to the high number of animals in this type of production [20]. In the case of ruminants, biosecurity measures have been implemented mainly on intensive dairy or meat production farms, characterized by the high number of animals within a delimited space without contact with animals from other similar farms [21,22]. However, implementing biosecurity measures in extensive livestock systems, particularly in less-favored regions, presents significant challenges. Factors such as the advanced age of farmers, lack of training, small herd sizes, grazing on communal lands, and the need for economic investment can hinder adoption. The mandatory implementation of biosecurity measures may even threaten the viability of some farms, potentially leading to the cessation of livestock activities [23]. This could have far-reaching consequences for the economic, social, and environmental sustainability of Portugal’s interior regions [24].

Given these challenges, this study aims to evaluate the biosecurity measures applied in small ruminant farms in Portugal, focusing on their role in ensuring the economic, social, and environmental sustainability of these regions. This research is particularly relevant as it is the first to focus on biosecurity in small ruminant farms in Portugal, filling a gap left by previous studies that primarily concentrated on dairy cattle, swine, and poultry [25]

## 2. Materials and Methods

### 2.1. Biosecurity Assessment

A cross-sectional study of biosecurity knowledge and practices was conducted from 1 July 2023 to 1 April 2024. A voluntary survey (comprising personal interviews) was administered to 276 small ruminant farmers. A total of 300 farmers were previously contacted through the local livestock farmers’ organization to schedule an interview. Then, the objective of this study was explained in a personal interview, and after voluntary acceptance (with a signed informed consent form), the farmers were interviewed. To assess the knowledge about biosecurity, a specific checklist comprising 32 questions divided into 9 groups was designed based on the biosecurity checklist proposed by the Confederation of Portuguese farmers and a literature review [23,26,27]: (1) physical protection measures, (2) cleaning and disinfection, (3) facilities, (4) pest control, (5) animal farm management, (6) feed and water supply, (7) visitor’s control, (8) dead animal management and (9) disease control. Also, the sociodemographic characterization and biosecurity knowledge and perception of respondents were recorded.

Farm characterization includes information about flock size (classified into the following categories: ≤50 heads, <50 ≤ 100 heads, <100 ≤ 150 heads, <150 ≤ 200 heads, <200 ≤ 250 heads, <250 ≤ 300 heads, and >300 heads), main animal production (dairy, meat, or both), species (sheep, goats, or both), and location (see Supplementary Information SI).

Farms were classified as dairy or meat when the farmer reported in the personal interview that more than 50% of the animals were destined for dairy or meat production, respectively. Farms classified as mixed included those where the farmer indicated during the personal interview that half of the animals were used for meat production and the other half for dairy production, as well as those with two separate production units that belonged to the same farm. Regarding species classification, farms were categorized as sheep or goat farms when they exclusively housed animals of a single species. Farms were classified as mixed when they had at least one animal of each species.

To assess the clarity of the questionnaire instructions, layout, and time requirement, a pilot study was conducted on 25 small ruminant farmers. The results of this pilot study are not included in the Section 3.

The participants’ overall knowledge of biosecurity was assessed using 32 closed questions (yes or no). Questions answered with “yes” were assigned a value of 1, while questions answered with “no” were assigned a value of 0. The responses to these questions were combined to generate a knowledge score ranging from 0 to 32. To reduce the possibility of farmers answering correctly by chance, all questions included the item “I do not know”, which was also assigned a value of 0. According to the number of correct questions answered, the farmer’s knowledge about biosecurity was classified into 4 groups as follows:

- ‘‘Not satisfactory’’ (0–49%), characterized by scarce knowledge about animal biosecurity that may compromise the health status of the flock, policy compliance, and public health.
- ‘‘Satisfactory” (50–74%), meaning that the farmer knows the main characteristics of animal biosecurity but they apply/implement some measure in their flocks.
- ‘‘Acceptable’’ (75–89%), meaning that the farmer displayed an average knowledge of animal biosecurity and applied/implemented several measures in the flock.
- ‘‘Recommended’’ (90–100%), meaning that the farmer displayed a high level of knowledge and has already implemented a biosecurity plan in their flock.

Before completion, all farmers were informed of its voluntary and anonymous nature and its objectives. No sensitive data were collected. Since the surveys were conducted in person, data were only collected from those farmers who gave their consent for this purpose.

### 2.2. Data Analysis

All questionnaire data were entered into an Excel spreadsheet (Microsoft^®^ Excel^®^ for Microsoft 365, Redmon, WI, USA, version 2502) and carefully checked. Statistical analysis was conducted using JAMOVI ^®^ for Windows (version 19.0). The influence of the social characteristics of farmers on biosecurity knowledge or farm management was assessed by Kruskal–Wallis. Values with *p* < 0.05 were considered as statistically significant. Statistically significant variables resulting from the Kruskal–Walls test were subjected to pairwise comparison by the Dwass–Steel–Critchlow–Fligner test.

## 3. Results

### 3.1. Sociodemographic and Farm Characterization

Of the total of 300 ranchers contacted, only 276 were interviewed in person (response rate 92%), with most of them being men (Table 1). However, farm tasks were carried out by both men and women (59.1%). Respondents were generally older, with almost 64% (n = 176) being over the age of 65 and about 25% being between 46 and 64 (n = 81). Most of the respondents had only completed compulsory schooling (93.8%), although slightly more than half reported having attended training courses on various aspects of livestock production. Small ruminant livestock production (Table 2) is primarily focused on sheep for meat production. Most flocks are small (less than 50 heads) and are predominantly located in northern Portugal, whereas the largest flocks are found in the Alentejo region (Supplementary Table S1).

### 3.2. Biosecurity Knowledge and Evaluation of Biosecurity Measures at Farm

Knowledge of the concepts of biosecurity and biocontainment (Table 3) by farmers is scarce (75% and 90% respectively), which explains the response of the farmers interviewed that the implementation of biosecurity measures on the farm has no impact on its management.

The study of the different measures applied on farms to ensure biosecurity is indicated in Table 4. Since the number of questions varies among the nine groups (i.e., categories of biosecurity measures), no classification by group of measures was performed.

**Figure 1:**
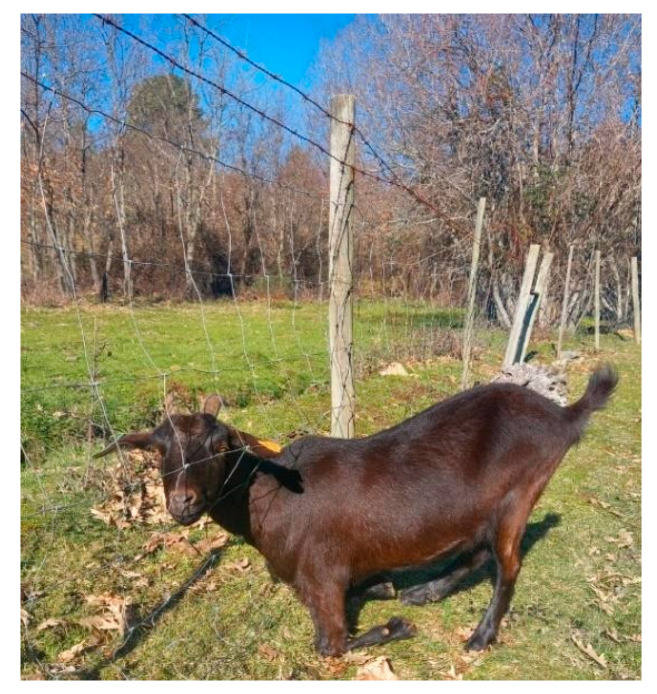
Fence trespassing due to lack of maintenance.

Regarding physical protection measures, most farms have livestock fenced by metal nets, although almost 75% of respondents indicated that maintenance is not adequate, which allows potential contact with other animals (74.6%), both domestic and wild (Figure 1).

Regarding cleaning and disinfection, the majority of respondents indicated that they do not have a documented cleaning and disinfection program (70.7%), although this is defined in most dairy farms (meat and milk: 79.7%, milk: 90.6%). Cleaning and disinfection operations on meat farms are generally carried out twice a year, coinciding with the removal of manure.

However, respondents indicated that they pay more attention to the hygiene of the animals’ feeders and drinkers.

**Figure 2:**
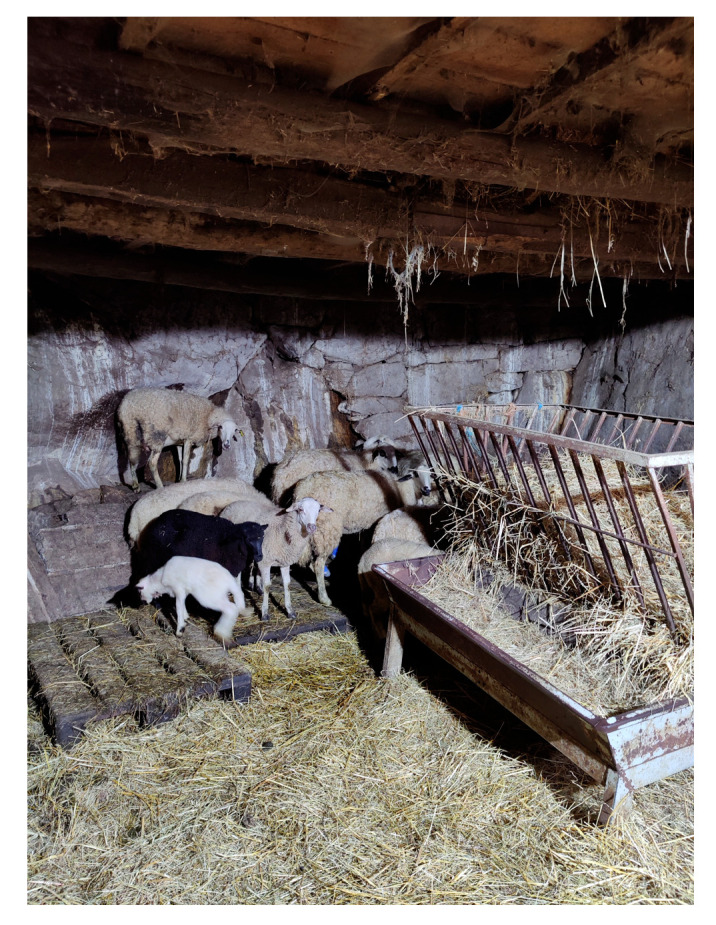
Traditional premises with a lack of hygienic design in the walls and ceiling.

Farm facilities in general do not usually have a hygienic design that facilitates cleaning and disinfection processes (Figure 2), with the most frequent hygienic design being in dairy farms (56.0%). Most farms do not have specific areas such as a maternity ward, lamb/kid area (No: 176 meat, 39 M&D, 16 milk; Yes: 9 meat, 20 M&D, 16 milk) or an isolation area for sick animals. However, almost 75% of farms with more than 101 heads have an area for sick animals. Regarding the existence of a vector control plan against insects or rodents, it is also rare, except for dairy farms.

**Figure 3:**
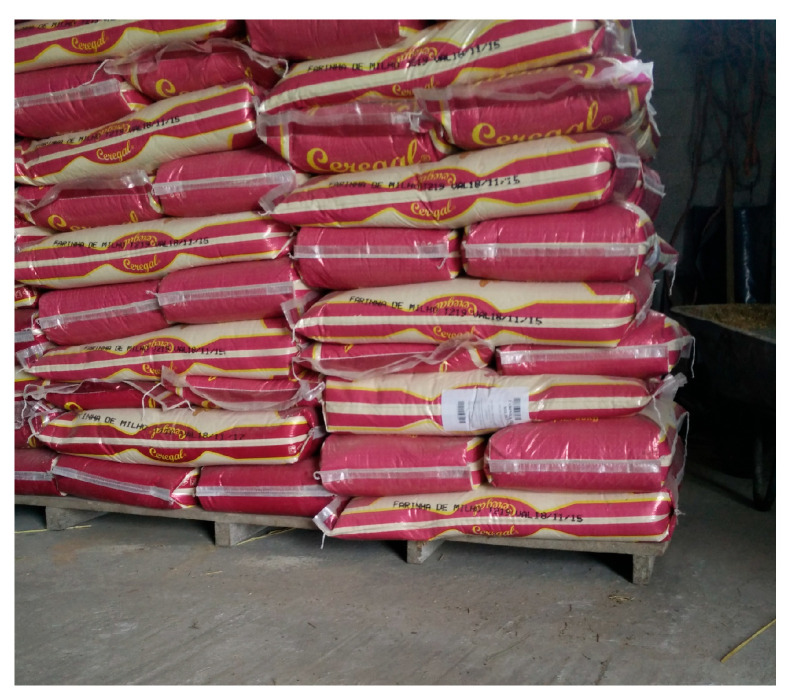
Proper feed storage.

Regarding animal management, most of the respondents showed knowledge about the animal movement policy (86.6%), although most of them stated that they perform replacement. Although almost all respondents know the rules on animal identification (96.0%), only a few farmers record the loss of ear tags (7.2%). Feed is mainly based on pasture, as most farms are extensively managed, although supplementation with concentrate is a common practice. Thus, most farmers are careful with storage conditions (Figure 3). However, traceability records cannot always be guaranteed. Regarding water supply, laboratory control is not a common practice. Livestock health management is mainly based on prophylactic treatments. Thus, the majority of respondents indicated that deworming treatments are carried out regularly (79.0%), whereas only 35.5% implement vaccine prophylaxis. In the case of sick animals, only 25% of respondents separate them from the herd, which is consistent with the lack of specific areas on the farm for this purpose, as mentioned above. Furthermore, the lack of a specific isolation area aligns with the high rate of respondents (82.6%) who declared that they do not implement quarantine schemes.

### 3.3. Biosecurity Score, Influence Factors, and Biosecurity Classification

The scores for the implementation of the different biosecurity measures (Table 5) of the farms in this study ranged from 15.1% to 69.7%. Overall, 90.2% of the farms were classified as unsatisfactory, while only 9.8% had satisfactory biosecurity measures in place. Among the farms classified as unsatisfactory, 90% have a low implementation of the different biosecurity measures (18.1–39.49%). However, almost 60% of the farms classified as satisfactory obtained compliance scores of just over 50% (51.5–54.5%). None of the farms achieved scores for the implementation of biosecurity measures classified as “acceptable” or “recommended”.

Table 6 presents the risk factors influencing biosecurity compliance scores in small ruminant farms. The most significant factor was herd size with larger farms demonstrating higher compliance. Age showed a moderate influence, particularly distinguishing older farmers from younger groups. Regarding production type, the results indicated that dairy and mixed farms had higher compliance than meat-only farms. Although specific training had no significant impact (*p* > 0.05), higher education levels have correlated with better biosecurity practices.

Regarding age (Table 7), most groups do not show significant differences. However, the “>66” age group differs significantly from the “36–45” and “56–65” groups, indicating possible differences in biosecurity compliance score. The effect size (ε^2^) suggests a small to moderate influence. Although difficult to explain, this could be associated with schooling and/or training. The biosecurity compliance score was also influenced by farmers’ education (Table 8), with there being differences between farmers with basic education and primary education as well as between farmers with high school and farmers with primary education. The relatively high significance (*p* < 0.001) indicates a strong association, although the effect size is slightly smaller compared to age.

Herd size has the largest chi-square value and effect size among the factors tested, indicating a very strong and significant influence on compliance scores (Table 9). Based on the herd size, significant differences were observed between the smallest groups (“≤50” and “51–100”) and the largest groups (“101–150” and “151–200”). This suggests that farms with smaller numbers of animals may have lower scores regarding the implementation of biosecurity measures. Furthermore, the statistically significant differences between the groups “≤50” and “>301” indicate that the smallest farms (“≤50”) and slightly larger ones (“51–100”) differ significantly from farms with “more than 301” heads, where the scores regarding the implementation of biosecurity measures are higher. Thus, herd size stands out with the highest chi-square statistic and effect size, indicating that it is a primary factor of differences in biosecurity compliance.

Regarding the type of production, the results (Table 10) suggest that biosecurity compliance score is more similar between meat and milk and milk-only farms, while meat-only farms stand out as different.

## 4. Discussion

Biosecurity measures are essential for preventing and controlling diseases that can affect sheep and goats, protecting public health and the environment. In the current One Health context, biosecurity on livestock farms plays a crucial role in protecting animal, human, and environmental health. With increasing risks of emerging and re-emerging diseases, including zoonotic diseases, antimicrobial resistance, and global trade, the implementation of biosecurity measures helps prevent disease outbreaks, ensuring food safety and food security, as well as contributing to economic and farming community sustainability. Aspects such as farm limits, cleaning and disinfection, controlled access, prophylactic veterinary schemes, and early disease detection reduce the transmission of pathogens between animals and humans. Strengthening biosecurity measures on farms is essential for sustainable livestock production and safeguarding public health, reinforcing the connection of animal, human, and environmental well-being.

Despite the importance of biosecurity [27] in the health management of livestock farms, research on the application of these measures remains very scarce [13]. Thus, to the best of the authors’ knowledge, this work is the first carried out in Portugal on small ruminants. Biosecurity plans must be adapted to each farm, and although there are some common measures [28] that can be implemented in all livestock farms, there is no scientific consensus on which measures should be included in these biosecurity plans [29].

The sociodemographic characterization of the livestock farms under study reveals an aging of livestock farmers, with little generational change [30]. Traditionally, livestock farming has been an activity dominated by men; however, in recent years, the participation of women in farm tasks has become increasingly frequent. This change can be explained using the classification of farms suggested elsewhere [31], in which peasant farms, characterized as old, traditional farms, managed by experience and with a low level of education (these being the main type of small ruminant livestock farms in Portugal), have a significantly high percentage of female labor. Thus, the education of farmers was also perceived as the most important and effective measure for protecting small ruminant farms from disease [32].

The knowledge of respondents on biosecurity is quite low, as reported elsewhere [33] which is consistent with their age, lack of education, and the type of production system [small-scale family farms] [23,24,34,35]. Thus, a higher level of knowledge on biosecurity is associated with a higher level of education of the livestock farmers interviewed, as has been reported [15,36]. Also, the scarce knowledge about biocontainment has been described elsewhere [37] and evidenced in an outbreak of sheep pox [38].

The low economic performance of these livestock farms also justifies the high percentage of respondents that declared biosecurity has no impact on the farm, whether in productive aspects [39,40] or in management aspects [37,41]. In contrast, those small ruminant farms with large flocks and better human, economic, and management resources displayed higher scores regarding biosecurity compliance and a higher number of animals [42].

On the other hand, the presence of a veterinarian seems to be an influential factor both in the adoption and/or implementation of biosecurity measures and as a source of technical information [40,41]. Thus, in dairy farms, due to their greater economic resources, the presence of a veterinarian is considered the most practical measure for implementing and improving biosecurity on the farm [43].

Most respondents indicated that they only contact a veterinarian when necessary, which could also explain the low level of knowledge on biosecurity observed.

Physical protection measures aim to avoid contact between farm animals and others to prevent disease transmission [13]. This measure is easy to comply with in intensive farms, mainly in dairy farms where keeping a closed herd was rated as the most effective measure overall [43]. Most of the respondents have their pastures fenced with hard wire mesh in good condition [17]. Although most of them indicate that external animals, both domestic and wild, can be vehicles of pathogenic agents [44,45], the main concern is the protection of the herd against attacks by predators (i.e., dogs or wolves), with hard wire mesh being an effective protection measure [46]. However, given the main type of production indicated by the respondents, contact with other animals [external vectors], both domestic and wild, is inevitable [47] since most of the herds share the same paths from the stable to the private pastures or common grazing areas. Although fencing is considered an important measure by the interviewees, its limited usefulness in the main type of farms [i.e., small-sized and extensive farms] studied has already been observed by [24]. Control of farm visitors has been considered an important biosecurity measure; however, the facilities’ design on most farms visited, together with the extensive management, explains the low value given by respondents [48]. Traditional livestock farm infrastructure, often built with materials such as wood and stone, lacks designated access points and visitor control measures, making it difficult to restrict access and implement biosecurity protocols. Furthermore, many farms lack separate entrances for staff and visitors, leading to uncontrolled movement and increasing the risk of disease transmission. Furthermore, small ruminant meat farms are characterized by the use of shared grazing areas, which makes visitor control even more difficult by reducing the effectiveness of physical barriers. Therefore, reinforcing facility design by incorporating controlled access points and designated visitor areas could improve biosecurity measures [49]. However, other authors reported higher control of personnel entry–exit frequency [47].

Maintaining a hygienic environment within farm facilities provides better conditions for animal health and welfare. However, the results obtained indicate that the implementation of cleaning and disinfection (C&D) programs is limited, these being more common in dairy farms [43,50]. This can be explained by the need for stricter hygienic control during milking operations. It has been observed [51] that as the size of small ruminant farms increases, the general hygiene of the facilities, particularly dairy farms, is more satisfactory. Contrary to what was observed, a higher frequency of disinfection procedures has been described in goat farms [52]. This can be explained by the fact that in farms with a large number of animals (mainly in dairy production), C&D is closely related to the prevention of diseases such as mastitis or foot problems, which can cause significant production losses [53]. The fact that, in the farms studied, C&D is limited to keeping the bedding dry and removing manure only twice a year has also been reported in other studies [47,50,53,54]. In addition, the lack of a hygienic design of the premises [i.e., mainly made from wood and stone] represents a barrier to the implementation of C&D plans [34], unlike what was observed in other studies, where almost all the small ruminant farms evaluated showed a satisfactory level of hygiene [50,55]. However, the perception of the respondents regarding the need to keep feeders and drinkers clean coincides with the findings of other authors [40,56].

Regarding the design of livestock farms, there are no specific regulations on the type of areas that should be included (e.g., quarantine areas, areas for sick animals, or areas for lambs/kids). However, sectoral guidelines recommend them [57,58,59,60,61,62,63]. The importance of these areas is due to the need to create barriers to prevent the introduction of pathogens or their spread, in the event of an outbreak [64]. The scarce existence of differentiated areas observed is compatible with the type of farm facilities, although a high rate of quarantine and isolation of sick animals has been reported [54]. Quarantine zones’ presence has been considered as one of the top five biosecurity measures [11]. However, for small ruminants, their existence is rare [12,48,50,56], although this practice seems to be more frequent in farms that have experienced disease outbreaks [65,66].

Moreover, the health control of incoming animals (i.e., quarantine) not only prevents the transmission of diseases to the existing livestock on the farm but also reduces the need for antimicrobial treatments, thereby helping to mitigate the rise of antimicrobial resistance [60].

While quarantining animals is essential, this practice is not particularly effective in controlling diseases such as paratuberculosis or maedi-visna, so other biosecurity measures must be implemented [34,67,68]. However, the transmission of diseases with a long incubation period has been perceived to be a risk of major significance in Europe [63]. The fact that there are livestock farmers who are aware of the existence of insidious and chronic diseases has led to the request for a health certificate related to maedi-visna or scrapie [12]. However, transmissible diseases are perceived in Europe to be of major significance from a conservation perspective [69].

A low rate of compliance regarding footbaths and ditches at the main entrance has also been reported [50,54], in accordance with our results.

Although farm management practices vary from farm to farm, respondents demonstrate a high level of knowledge on aspects such as identification and movement regulations, which are related to biosecurity. This suggests that farmers are aware that the uncontrolled entry of animals into the farm can pose not only a health risk but also a legal one [7,44,51].

Furthermore, the high number of respondents who prioritize breeding over purchasing animals is aligned with the perceived risk associated with the introduction of new animals [54,65]. Furthermore, it has been observed that when farmers purchase animals, they make an effort, as far as possible, to verify the biosecurity status of the farm from which the animals come [69,70]. Although identification has been defined as a key factor in farm biosecurity, some farmers have expressed opposition to electronic identification [71].

Feed and water monitoring is essential to ensure animal health and food safety. Feed must be stored under hygienic conditions to avoid contamination by external vectors [12]. Although almost half of the respondents perceive the need for adequate storage, other aspects, such as water quality control and traceability record keeping, remain undervalued, as previously reported [34,50].

Farm visits may represent a risk of transmission of pathogens to animals, although the probability is low. The limited biosecurity measures observed in this area [registration of entries and exits, use of personal protective equipment] are in agreement with other studies on small ruminant farms [34,48]. Although other authors have reported the existence of controls and registration of entries and exits, the implementation of measures such as the use of PPE has been scarce [50]. Additionally, hesitation among farmers to request that visitors use PPE has been reported [71].

Deaths on a livestock farm represent not only an economic loss but also a health and environmental risk. Thus, their disposal must be rapid and safe. Respondents demonstrated a good knowledge regarding the importance of prompt and proper disposal of carcasses compared to other studies [34,72]. However, the presence of a dedicated carcass storage facility [e.g., leak-proof containers] appears to be limited across small ruminant farms [12].

Prophylactic measures are essential for the prevention of infectious and parasitic diseases. Almost 95% of respondents deworm their herds [7,51,73], although vaccine prophylaxis is less common [52], either due to a lack of knowledge of the recommended vaccines or insufficient veterinary technical support [74,75]. In addition, a high rate of self-treatment of animals by farmers contributes to the lack of contact with veterinarians and, consequently, better information on biosecurity measures applied to infectious diseases [76]. Other authors indicate that the level of knowledge of farmers on routine measures for preventing the introduction of diseases is adequate, although these works do not evaluate their level of knowledge on the main diseases that affect small ruminants [48,77].

The assessment of biosecurity compliance on livestock farms is a challenge, as there are no specific standards for each type of production, nor is there a consensus on the specific measures that should be included [29]. A lower score regarding biosecurity compliance has also been reported [33]. In addition, research on biosecurity on small ruminant farms is scarce, and the parameters used in each of the studies to obtain a classification regarding biosecurity compliance are not homogeneous among them. The results indicated that only 10% of the farms studied implement biosecurity measures satisfactorily, a value significantly lower than that reported in other studies [65,78]. Also, it was observed that factors such as age, education, herd size, and type of production seem to influence the biosecurity compliance level. However, these factors should be considered with some caution given the local characteristics of small ruminant farms and their management since other authors have not observed differences in compliance rates based on age, education, or professional experience [54].

It seems that the degree of implementation of biosecurity measures is higher among farmers aged between 35 and 65 years, as in our study. Although difficult to justify, this could be associated with higher levels of education and greater access to information through the Internet [33,79]. Herd size and production type also seem to influence biosecurity compliance scores, as farms with a high number of animals and/or dairy farms tend to have better infrastructure and equipment, which facilitate the implementation of various biosecurity measures [80,81,82].

## 5. Conclusions

Biosecurity in livestock production is essential for animal health, public health, and product quality. This study evaluated biosecurity practices on small ruminant farms in Portugal, revealing a low level of implementation. Only 9.8% of farms received a “satisfactory” rating, and none met higher standards.

Demographic analysis showed that an aging livestock farmer population and low education levels hamper the implementation of biosecurity plans. Contributing factors include lack of training, small-scale family production, low profitability, and limited veterinary support. Larger farms and dairy farms showed greater biosecurity compliance.

Farmers primarily implemented biosecurity measures related to animal identification and movement, while cleaning and disinfection, visitor control, and external vector management were largely neglected. Furthermore, inadequate farm infrastructure, including the absence of areas such as quarantine, farrowing, isolation for sick animals, or carcass storage, further compromised disease prevention.

Thus, the low score observed regarding the implementation of biosecurity measures may be related to specific farm and management characteristics, such as an extensive, small-scale, and family-run production system, coupled with an aging farmer population, low education levels, and economic constraints, which significantly hinder the adoption of biosecurity measures.

These findings highlight the urgent need for livestock farmer training, improved veterinary support, and biosecurity strategies tailored to extensive livestock farming systems. Public policies must provide incentives and educational programs to improve biosecurity without compromising farm viability. Ensuring the effective implementation of biosecurity plans requires collaboration between farmers, veterinarians, and authorities to protect animal and public health while supporting the sustainability of rural communities in Portugal.

It is important to highlight that the lack of standardized biosecurity criteria for small ruminant production, making it difficult to compare results with other studies and establish clear compliance benchmarks, represents a limitation. Moreover, future research should focus on developing standardized biosecurity assessment schemes. In addition, an investigation into the role of veterinary services and farmer training programs in improving biosecurity compliance is necessary.

## Figures and Tables

**Table 1:** Sociodemographic characterization of the respondents.

		n	%
Animal and farm operation			
	Men	82	29.7
	Women	31	11.2
	Both	163	59.1
Age			
	<25	2	0.7
	26 ≤ 35	7	2.5
	36 ≤ 45	10	3.6
	46 ≤ 55	62	22.5
	56 ≤ 65	19	6.9
	≥65	176	63.8
Level of education			
	Primary school	259	93.8
	High school	17	6.2

**Table 2:** Farm characterization.

			n	%				n	%			n	%	
Location														
	North		212	76.8		Centre		48	17.4					
		Goats	62	22.5			Goats	21	7.6					
		Sheep	119	43.1			Sheep	16	5.8					
		Both	31	11.2			Both	11	4.0					
	Alentejo		15	5.4		Algarve		1	0.4					
		Goats	2	0.7			Goats	1	0.4					
		Sheep	12	4.3			Sheep	0	0.0					
		Both	1	0.4			Both	0	0.0					
Farmers’ specific training in animal health and/or management														
	Yes		155	56.2										
	No		121	43.8										
Main species														
	Goats		86	31.2		Sheep		147	53.3	Both		43	15.5	
		Mean	58	-			Mean	50.8	-		Mean	96		
		Minimum	1	-			Min.	1	-		Min.	9		
		Maximum	600	-			Max.	780	-		Max.	650		
Herd size														
≤50 heads			216	78.3										
	Species					Location								
		Goats	65	23.6			Norte	190	68.8					
		Sheep	125	45.3			Centro	20	7.2					
		Mixed	26	9.4			Alentejo	6	2.2					
							Algarve	0	0.0					
<50 ≤ 100 heads			21	7.6										
	Species					Location								
		Goats	6	2.2			Norte	13	4.7					
		Sheep	8	2.9			Centro	8	2.9					
		Mixed	7	2.5			Alentejo	0	0.0					
							Algarve	0	0.0					
<100 ≤ 150 heads			11	4.0										
	Species					Location								
		Goats	4	1.4			Norte	4	1.4					
		Sheep	5	3.3			Centro	5	1.8					
		Mixed	2	4.0			Alentejo	1	0.4					
							Algarve	1	0.4					
<150 ≤ 200 heads			10	3.6										
	Species					Location								
		Goats	6	2.2			Norte	4	1.4					
		Sheep	1	0.4			Centro	6	2.2					
		Mixed	3	1.1			Alentejo	0	0.0					
							Algarve	0	0.0					
<200 ≤ 250 heads			2	0.7										
	Species					Location								
		Goats	1	0.4			Norte	0	0.0					
		Sheep	0	0.0			Centro	2	0.7					
		Mixed	1	0.4			Alentejo	0	0.0					
							Algarve	0	0.0					
<250 ≤ 300 heads			3	1.1										
	Species					Location								
		Goats	1	0.4			Norte	1	0.4					
		Sheep	1	0.4			Centro	0	0.0					
		Mixed	1	0.4			Alentejo	2	0.7					
							Algarve	0	0.0					
>300 heads			13	4.7										
	Species					Location								
		Goats	3	1.1			Norte	0	0.0					
		Sheep	7	2.5			Centro	7	2.5					
		Mixed	3	1.1			Alentejo	6	2.2					
							Algarve	0	0.0					
Main animal production														
	Meat		202	73.2		Meat and Dairy		59	21.4	Dairy			15	5.4
		Goats	62	22.5			Goats	18	6.5		Goats		6	2.2
		Sheep	113	40.9			Sheep	26	9.4		Sheep		8	2.9
		Mixed	27	9.8			Mixed	15	5.4		Mixed		1	0.4
		Alentejo	7	2.5%			Alentejo	6	2.2		Alentejo		2	0.7
		Algarve	1	0.4%			Algarve	0	0.0		Algarve		0	0.0
		Centro	13	4.7%			Centro	31	11.2		Centro		4	1.4
		Norte	181	65.6%			Norte	22	8.0		Norte		9	3.3
Veterinary support at farm														
	Employed *		30	10.9										
	When necess.		246	89.1										

Min.: minimum; Max: maximum; M&M: meat and milk; necess: necessary; *: refers to farm’s staff.

**Table 3:** Biosecurity knowledge.

Knowledge about the Concept of Biosecurity								
		n	%				n	%
Yes		69	25.0		No		207	75
	Primary school	54	19.6			Primary school	205	74.2
	High school	15	5.4			High school	2	0.8
Knowledge about the concept of biocontainment								
Yes		28	10.2		No		248	89.8
	Primary school	14	5.0			Primary school	245	88.7
	High school	14	5.0			High school	3	1.1
Main reasons for implementing (or not) a biosecurity plan on the farm								
	Improve the animal welfare						10	3.6
	Improve the quality and safety of products						9	3.3
	Increase the economic performance of the farm						40	14.5
	The implementation of biosecurity measures has no impact on the management of the farm						217	78.6

**Table 4:** Evaluation of biosecurity measures at farms.

							n	%
Physical protection measures								
	Farm limits. Type of fencing							
		Barbed wire					2	0.7
		Electric wire					2	0.7
		Hard wire mesh					223	80.8
		Wall					49	17.8
	The fence is in good condition							
		Yes					76	27.5
		No					200	72.5
	Potential contact with wildlife							
		Yes					202	73.2
		No					74	26.8
	Existence of separate entrances to the farm for staff and visitors							
		Yes					5	1.8
		No					271	98.2
Cleaning and disinfection								
	Existence of a cleaning and disinfection program							
		Yes					81	29.3
		No					195	70.7
	Proper cleaning and disinfection of infrastructures and/or equipment							
		Yes					148	53.6
		No					128	46.4
	Frequency of cleaning of premises							
		Weekly					15	5.5
		Monthly					26	9.4
		Every three months					4	1.4
		Twice a year					205	74.3
		Once a year					26	9.4
	Frequency of disinfection of premises							
		Weekly					9	3.3
		Monthly					17	6.2
		Every three months					3	1.1
		Twice a year					155	56.2
		Once a year					41	14.9
		No disinfection applied					51	18.5
	Maintenance of animals’ feeders, proper cleaning and disinfection							
		Yes					248	89.9
		No					28	10.1
	Cleaning and disinfection of work clothes							
		Yes					107	38.8
		No					169	61.2
Facilities								
	Floor, ceiling, and walls designed for easy cleaning and disinfection							
		Yes					72	26.1
		No					204	73.9
	Existence of a maternity area							
		Yes					45	16.3
		No					231	83.7
	Existence of an area for kids and/or lambs							
		Yes					47	17.0
		No					229	83.0
	Existence of a specific area for sick animals							
		Yes					45	16.3
		No					231	83.7
	Existence of footbaths							
		Yes					72	26.1
		No					204	73.9
	Existence of wheel washes							
		Yes					12	4.3
		No					264	95.7
Pest control								
	Windows with mosquito net							
		Yes					29	10.5
		No					247	89.5
	Existence of rodent control program							
		Yes					24	8.7
		No					252	91.3
	Existence of insect control program							
		Yes					23	8.3
		No					253	91.7
Animal farm management								
	Record of ear tag loss							
		Yes					20	7.2
		No					256	92.8
	Knowledge of animal movement policy							
		Yes					239	86.6
		No					37	13.4
	Knowledge about animal identification policy							
		Yes					265	96.0
		No					11	4.0
	Purchase of animals							
		Yes					56	20.3
		No					220	79.7
Feed and water supply								
	Keep feed protected from pests							
		Yes					126	45.7
		No					150	54.3
	Record of feed traceability							
		Yes					16	5.8
		No					260	94.2
	Laboratory control of water supply for animals							
		Yes					35	12.7
		No					241	87.3
Visitor’s control								
	Record system of farm visitors							
		Yes					13	4.7
		No					263	95.3
	Existence of specific PPE for visitors							
		Yes					273	98.9
		No					3	1.1
Dead animal management								
	Type of death animal removal							
		Mandatory animal burial					214	77.5
		Official collection of dead animals					62	22.5
	Existence of measures to prevent farm animal contact with dead animals							
		Yes					266	96.4
		No					10	3.6
	Existence of specific place to store dead animals							
		Yes					49	17.8
		No					227	82.2
Disease control								
	Application of quarantine protocols							
		Yes *					48	17.4
		No					228	82.6
	Existence of a vaccination schedule for herd/flock							
		Yes					98	35.5
		No					178	64.5
	Existence of a deworming schedule for herd/flock							
		Yes					218	79.0
		No					58	21.0
	Frequency of animal deworming							
		Once a year					69	31.5
		Twice a year					141	64.4
		Three times a year					5	2.3
		Four times a year					4	1.8
	Isolation of sick animal							
		Yes					70	25.4
		No					206	74.6
	Knowledge of the sanitary status of the herd							
		Yes					165	59.8
		No					111	40.2
	Existence of contingency plan for disease outbreak at farm							
		Yes					8	2.9
		No					268	97.1
Manure management								
	Frequency of manure removal							
		Once a week					15	5.4
		Once a month					15	5.4
		Three times a year					16	5.8
		Twice a year					192	69.6
		Once a year					38	13.8
	Destination of manure							
		Crop fertilization					276	100

* Only includes those farmers who declared “not purchase animals”.

**Table 5:** Biosecurity compliance score.

Biosecurity Score ^a^	Biosecurity Classification	Farms ^b^	% ^c^
15.15	Non-satisfactory	7	2.5%
18.18	Non-satisfactory	18	6.5%
21.21	Non-satisfactory	21	7.6%
24.24	Non-satisfactory	33	12.0%
27.27	Non-satisfactory	39	14.1%
30.30	Non-satisfactory	41	14.9%
33.33	Non-satisfactory	28	10.1%
36.36	Non-satisfactory	21	7.6%
39.39	Non-satisfactory	20	7.2%
42.42	Non-satisfactory	9	3.3%
45.45	Non-satisfactory	7	2.5%
48.48	Non-satisfactory	5	1.8%
51.51	Satisfactory	6	2.2%
54.54	Satisfactory	10	3.6%
57.57	Satisfactory	4	1.4%
60.60	Satisfactory	2	0.7%
63.63	Satisfactory	2	0.7%
66.66	Satisfactory	1	0.4%
69.69	Satisfactory	2	0.7%

^a^: The biosecurity compliance score ranged from 0 to 100%. ^b^: Represents the total number of farms that displayed the same biosecurity compliance score. ^c^: % of farms.

**Table 6:** Risk factors influencing the biosecurity compliance score.

	χ²	df	*p*	ε²
Age	21.6	5	*p* < 0.01	0.0787
Education	16.7	2	*p* < 0.001	0.0606
Specific training	0.0803	1	*p* > 0.05	2.92 × 10^−4^
Herd size	62.3	6	*p* < 0.001	0.226
Production *	19.5	2	*p* < 0.001	0.0711

df: degrees of freedom. ε^2^: effect size. *: refers to meat or dairy.

**Table 7:** Comparison between ages.

Pair Comparison		w	*p*
≤25	≥66	−1.405	0.920
26–35	36–45	1.738	0.823
26–35	46–55	−0.340	1.000
26–35	56–65	1.600	0.869
26–35	≤25	0.416	1.000
26–35	≥66	−0.813	0.993
36–45	46–55	−3.988	0.054
36–45	56–65	0.686	0.997
36–45	≤25	−1.242	0.952
36–45	≥66	−4.819	0.009
46–55	56–65	3.390	0.157
46–55	≤25	1.046	0.977
46–55	≥66	−1.240	0.952
56–65	≤25	−1.193	0.959
56–65	≥66	−4.821	0.009

**Table 8:** Comparison between education levels.

Pair Comparison		w	*p*
Basic education	High school	−0.536	0.924
Basic education	Primary education	−4.458	0.005
High school	Primary education	−3.939	0.015

**Table 9:** Comparison between herd sizes.

Pair Comparison		W	*p*
101–150	151–200	0.853	0.997
101–150	201–250	1.822	0.858
101–150	251–300	0.887	0.996
101–150	51–100	−4.614	0.019
101–150	≤50	−5.667	0.001
101–150	≥301	0.537	1.000
151–200	201–250	1.989	0.799
151–200	251–300	0.121	1.000
151–200	51–100	−5.454	0.002
151–200	≤50	−6.251	<0.001
151–200	over 301	−0.177	1.000
201–250	251–300	−0.816	0.997
201–250	51–100	−3.290	0.231
201–250	≤50	−3.255	0.243
201–250	>301	−1.215	0.978
251–300	51–100	−3.508	0.166
251–300	≤50	−3.586	0.147
251–300	>301	−0.768	0.998
51–100	≤50	−1.772	0.873
51–100	≥301	4.931	0.009
≤50	≥301	6.403	0.001

**Table 10:** Comparison between types of production.

Pair Comparison		W	*p*
Meat	Meat and milk	5.566	<0.001
Meat	Milk	3.546	0.033
Meat and milk	Milk	−0.143	0.994

## Data Availability

Data are contained within the article and Supplementary Materials.

## Supplementary Materials

The following supporting information can be downloaded at https://www.mdpi.com/article/10.3390/vetsci12040334/s1: Table S1: supplementary information.
